# Immediate-Release Nifedipine Binary Dry Powder Mixtures with Nanocellulose Featuring Enhanced Solubility and Dissolution Rate

**DOI:** 10.3390/pharmaceutics11010037

**Published:** 2019-01-18

**Authors:** Athanasios Mantas, Albert Mihranyan

**Affiliations:** Nanotechnology and Functional Materials, Department of Engineering Sciences, Uppsala University, 75121 Uppsala, Sweden; athanasios.mantas@angstrom.uu.se

**Keywords:** amorphous solid dispersions, calcium channel blockers, biorelevant media, Cladophora cellulose

## Abstract

Nifedipine (NIF) is a 1,4-dihydropyridine-based calcium channel blocker with poor solubility, whose bioavailability is highly dependent on the type of formulation. Dry powder mixtures of 20% *w*/*w* NIF with microcrystalline cellulose (MCC) and its high surface area nanocellulose analogue, which is namely Cladophora (CLAD) cellulose, were produced by heating at the melting temperature of the drug for 1 h. Non-heated samples were used as a reference. The solid-state properties of the mixtures were characterized by scanning electron microscopy, differential scanning calorimetry and X-ray diffraction. The drug release was studied in biorelevant media, including simulated gastric fluid (SGF), fasted-state simulated intestinal fluid (FaSIF) and fed-state simulated intestinal fluid (FeSIF). An enhanced apparent solubility and faster dissolution rate of NIF were observed in the heated mixture of NIF with CLAD-H in all tested biorelevant media (i.e., SGF, FaSIF and FeSIF), which was due to NIF amorphization in the high surface area nanocellulose powder. Ordinary MCC, which is essentially non-porous, did not produce an enhancement of a similar magnitude. The results of the study suggest that dry powder formulation using high surface area nanocellulose is a facile new strategy for formulating calcium channel blocker drugs, which could potentially be a viable alternative to currently used soft gel liquid capsules.

## 1. Introduction

Nifedipine (NIF) is a BCS class II drug with solubility-limited bioavailability. Furthermore, it experiences substantial first-pass metabolism in liver (42–56%). It is mainly used to treat cardiovascular diseases, such as hypertensive crisis, angina pectoris and chronic hypertension [1]. The pharmacokinetics of NIF is highly influenced by its formulation. The first formulations of NIF were immediate-release (IR) soft gel capsules (SGC), followed by retarded action tablets and finally, extended-release osmotic pump tablets [2]. When the plasma concentration of NIF increases rapidly, e.g., by intravenous bolus injection or IR SGC, a rebound effect may occur, which is recognized by an increase in heart rate (29–38%) but little or no change in blood pressure [3,4,5,6]. During prolonged treatment, the heart rate is not significantly affected, while systolic and diastolic blood pressure is significantly reduced [7]. On the other hand, when NIF is administered in a sustained release formulation, the decrease in blood pressure is not accompanied by tachycardia. When choosing between IR and modified-release (MR) formulations, dose and daily intake frequency should be considered, i.e., IR formulations are prescribed in low 5–10 mg doses 3 times a day, while MR tablets are a twice-a-day or single high-dose formulation with a maximum allowable daily dose of 60 mg [8,9]. Nowadays, IR NIF capsules are normally used if no other treatment is appropriate. However, IR NIF capsules can still be used to treat Raynaud’s disease in patients without a history of hypertension or ischemic heart disease. The dose of IR formulations should not exceed 20 mg per tablet. In fact, the SGC and IR tablet formulations containing 20 mg of NIF as the only active ingredient have been withdrawn from the market in many countries to avoid possible adverse effects [10].

According to the biowaiver monograph for NIF IR formulations, NIF is not ionized in the pH range found in GIT and thus, its solubility is independent of the pH of the medium in this range [10]. After oral administration, there is rapid and complete absorption of NIF into the gastrointestinal tract (GIT). The most common IR dosage forms include SGCs and film-coated tablets. The rate of absorption from film-coated tablets is slower than that from SGCs, which is around 1.6 h for SGC and 4.2 h for tablets, respectively [11,12]. However, it should be mentioned that t_max_ for SGCs showed high variability, with values in the range of 0.25–2.9 h having been reported in other studies [10]. Normally, there is no significant difference in C_max_ and AUC between SGCs and film-coated tablets. 

In order to develop solid-dosage form alternatives to SGCs in the form of tablets, the issue of low NIF solubility needs to be addressed. Several strategies for improving the solubility of NIF have been employed in the past, including micronization [13], nanocrystals [14] and wet ball milling with surfactants [15]. An enhanced solubility of NIF was observed in hot-melt mixtures with PVP and PEG as well as in inclusion complexes with β-cyclodextrin [16]. It is further known that amorphous solid dispersions enhance the solubility of many poorly soluble drugs [17,18], which has also been adopted for NIF formulations [19,20]. In this article, the amorphization of NIF in mixtures with microcrystalline cellulose (MCC) and its high surface area analogue in the form of nanocellulose is explored.

MCC is a common excipient, which is used as a binder/diluent in tablets and capsules both by wet granulation and direct compression. Ordinary MCC is characterized by a relatively low surface area, i.e., ≤1 m^2^/g, but nanocellulose can provide much larger surface area, i.e., 60–100 m^2^/g [21,22]. Furthermore, nanocellulose can produce stronger tablets during direct compression compared to MCC [22,23] and it can be used as a carrier of liquid drugs [24]. Nanocellulose may also accelerate the rate of hydrolytic degradation of moisture-sensitive drugs, e.g., aspirin [25,26,27].

It has previously been shown that the dissolution rate of NIF can be significantly increased (30–37-fold) by hot-melt extrusion of multi-component mixtures with polyvinylpyrrolidone (PVP) or hydroxypropylcellulose (HPC), i.e., NIF-PVP-MCC or NIF-HPC-MCC, which was due to crystalline-to-amorphous drug transitions [28]. It should be noted that binary mixtures of NIF with PVP or HPC as well as NIF-MCC failed to produce an enhancement of a similar magnitude. The highest detectable solubility of NIF in these studies was observed to be 13 μg/mL in 0.1 N HCl [28]. Recently, amorphization of NIF-PVP directly in gelatin capsules using in situ freeze-drying with tert-butanol was shown to result in enhanced dissolution properties [29].

We have recently shown that the formulation of ibuprofen and other arylpropionic acid derivatives with nanocellulose can result in enhanced solubility and rapid dissolution rate due to amorphization of the drug [30]. Ordinary MCC, which has a low surface area, fails to produce this effect. In this article, we continue exploring the formulation of poorly soluble drugs from various pharmacological classes with high surface area nanocellulose. In particular, we demonstrate how the solubility and dissolution rate of NIF can be substantially increased in dry powder mixtures with high surface area nanocellulose.

## 2. Materials and Methods 

### 2.1. Materials

CLAD was provided by FMC Biopolymers (currently DuPont, Philadelphia, PA, USA). MCC (Avicel PH101) and nifedipine (NIF) was purchased from Sigma Aldrich (St. Louis, MO, USA) (≥98% purity). Biorelevant media of simulated gastric fluid (SGF), fasted-state simulated intestinal fluid (FaSIF) and fed-state simulated intestinal fluid (FeSIF) were prepared using powder purchased from biorelevant.com (London, England) according to the manufacturer’s instructions. All chemicals were used as received without further purification. Table 1 presents the chemical structure of NIF.

### 2.2. Mixture Preparation 

Since nifedipine is a photosensitive substance, all mixtures were placed in amber vials, which were additionally covered in aluminum foil for extra photoprotection. 

#### 2.2.1. Non-Heated Mixture 

The weight ratio between NIF and cellulose was 2:8. Typically, 20 mg of NIF was mixed with 80 mg of cellulose in a sealed glass vial with cap using a Turbula mixer (Muttenz, Switzerland) for 15 min at 72 rpm.

#### 2.2.2. Heated Mixture 

Following the preparation of the powder mixtures as described above, the sealed vials were heated to the melting temperature of NIF (i.e., 174 °C) for 1 h. It was ascertained that no thermal degradation of NIF occurred during the heat treatment, which is shown in Figure S1 in Supplementary Material. All samples were stored at room temperature from the time of preparation to allow for possible recrystallization and used after 24 h.

### 2.3. Methods

#### 2.3.1. Scanning Electron Microscopy (SEM)

The Zeiss Merlin FEG-SEM instrument (Jena, Germany) was used. Powder samples were mounted on aluminum stubs using double-adhesive carbon tape and further sputtered with Au/Pd to reduce charging effects. A sputter coater (Polaron, Ashford, UK) was used. The sputtering settings were 4 × 10^−2^ mbar and 35 mA and for a sputtering time of 30 s. 

#### 2.3.2. Differential Scanning Calorimetry (DSC)

The DSC measurements were performed with the DSC 3+ instrument (Mettler Toledo, Schwerzenbach, Switzerland). Nitrogen gas at a flow rate of 60 mL/min was utilized throughout the measurements. 

##### Cryoporometry

A total of 10 mg of cellulose was placed in 1.5-mL Eppendorf tubes. One milligram of de-ionized water was added and the tube was shaken rigorously. After that, the tube was left overnight at room temperature. On the next day, the tube was centrifuged at 20,000 rpm for 6 min. Water was decanted and any free drops were carefully removed with a piece of paper. The samples were placed in aluminum pans with a lid and first cooled from room temperature to 243.15 K (−30 °C) at a rate of 10 K min^−1^ before being heated to around 283.15 K (10 °C) at a heating rate of 0.7 K min^−1^. All measurements were obtained in triplicate.

The pore size was calculated according to Landry [31]:

(1) $$r_{p}=\left(\frac{19.082}{\Delta T+0.1207}\right)+1.12$$

where *r_p_* is the radius of pore (nm) and Δ*T* is the difference between the peak maximum for melting of pore-confined water and peak value for melting of bulk water.

##### Melting Enthalpy

The samples were carefully weighed and placed in aluminum pans with a lid. The samples were first cooled from room temperature to 233.15 K (−40 °C) before being heated to around 473.15 K (200 °C) at a heating rate of 10 K min^−1^. The average sample weight was 2.15 ± 0.03 mg. All measurements were obtained in triplicate.

The crystallinity index (*CrI*) of NIF was calculated as follows:

(2) $$CrI=\left(\frac{\Delta H_{mix}}{\Delta H_{NIF}\times a}\right)\times 100$$

where Δ*H_mix_* is the enthalpy of NIF melting in the mixture, Δ*H_NIF_* is the melting enthalpy of pure NIF and *a* is the correction factor corresponding to NIF content, i.e., *a* = 1 for pure NIF and *a* = 0.2 for 20% *w*/*w* mixture. 

#### 2.3.3. X-ray Diffraction (XRD)

An X-ray diffractometer (D8 Twin-Twin, Bruker, Karlsruhe, Germany) with Bragg-Brentano geometry (CuKα radiation; λ = 1.54 Å) was used. The operating current settings were 40 kV and 40 mA. The 2θ angle was varied between 10 and 45° at 0.02° scan steps. The data were collected on flat powders, which were placed in reduced background specimen holders supplied by the manufacturer (Bruker).

#### 2.3.4. In Vitro Dissolution Test in Biorelevant Media

The dissolution test was performed in SOTAX (AT7 Smart, Basel, Switzerland) apparatus using 500 mL of the biorelevant medium per dissolution vessel. The dissolution bath was covered with aluminum foil for extra photoprotection. The temperature for each dissolution vessel was maintained at 37 °C. The same amount of mixed samples was used in all timings and all different media (20 mg NIF + 80 mg cellulose). Each group of non-heated and heated samples was run in triplicate. The paddle speed for each dissolution vessel was 50 rpm. The sampling times for all media were 5, 15, 30, 45, 60, 90 and 180 min, respectively. At each time point, 5 mL of the medium was withdrawn and filtered through a 0.45-μm PTFE filter after discarding the first 2–3 mL. A total of 1.5 ml of the remaining sample was transferred in glass vials for further analysis by HPLC.

#### 2.3.5. High Performance Liquid Chromatography (HPLC)

The HPLC-UV system was optimized for the detection of NIF prior to the analysis. The liquid chromatography system used was a quaternary pump (Agilent, Waldbrohn, Germany) with an autosampler and Xbridge BEH C18, 3.5 μm, 2.1 × 50 mm, Waters column. The column temperature was 50 °C and injector temperature was 20 °C. The mobile phase A was 0.1% formic acid in water and mobile phase B was 0.1% formic acid in acetonitrile. The flow rate was 0.8 mL/min. The injector wash was 50% acetonitrile. The retention time was 4.45 min and run time was 8.0 min. UV detection at the wavelength of 343 nm was used. Calibration samples were run prior to the analysis of the study samples by preparing the NIF stock solution in the biorelevant medium at a concentration of 250 μg/mL. The stock solution was used as the calibration standard by a subsequent gradual dilution of 1:250. 

#### 2.3.6. Solid-State Stability

The normal and heated powder mixtures of NIF-CLAD were produced as described above and stored at 40 °C and 75% RH in a desiccator, which was equilibrated over a saturated NaCl solution. The solid-state stability of the mixtures was regularly monitored using the DSC instrument (Mettler Toledo, Schwerzenbach, Switzerland) to record melting enthalpies. The DSC procedure was performed as described above. 

## 3. Results

Prior to discussion of the observed results, it is important to note that the term amorphization that is used throughout the rest of the text is meant to indicate a loss of long-range periodicity that is an important characteristic of fully crystalline structures, while some short-range order may still be present.

### 3.1. SEM

**Figure 1:**
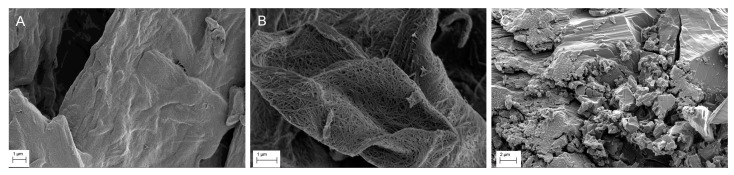
SEM images of pure MCC (**A**), CLAD (**B**) and NIF (**C**) samples.

Figure 1 shows the SEM topography images of pure cellulose and NIF samples. MCC and CLAD show different topographic structures as seen in Figure 1A,B. CLAD consists of numerous intertwined nanofibers, which cannot be seen in MCC. Figure 1C shows the typical topography of the NIF sample, which is useful for the discussion of the structural changes occurring in the mixtures with cellulose as outlined below.

**Figure 2:**
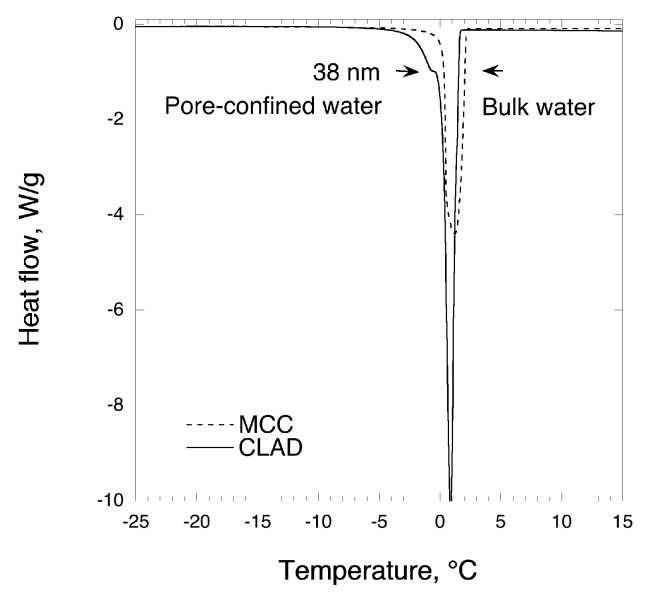
Cryoporometry with DSC of MCC and CLAD samples.

As shown in Figure 2, cryoporometry data confirm that the CLAD sample is much more porous than MCC since the pore-confined water peak is clearly detectable in this sample, which corresponds to a peak size of around 38 nm. These results corroborate with previously reported nitrogen gas sorption analysis data, suggesting that CLAD has a highly porous structured compared to the non-porous structure of MCC [30].

**Figure 3:**
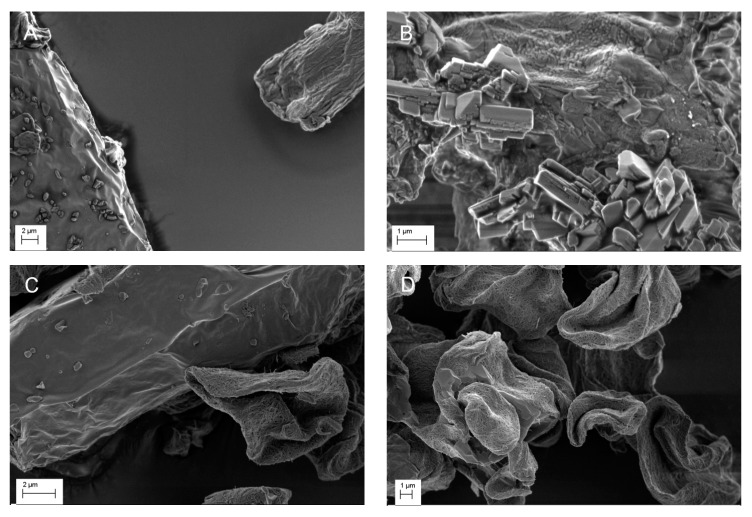
SEM images of NIF-MCC-N (**A**), NIF-MCC-H (**B**), NIF-CLAD-N (**C**) and NIF-CLAD-H (**D**) samples.

Figure 3 shows the SEM images of the NIF mixtures with cellulose samples. For non-heated mixtures, NIF crystals could be distinguished from the cellulose particles as separate particles, which is shown in Figure 3A,C. As shown in Figure 1B, the rough surface structures in the heated NIF-MCC mixtures were obviously reminiscent of those seen in pure NIF, suggesting that NIF covered the MCC surface. For the heated NIF-CLAD mixture, it is seen that NIF was incorporated inside the porous structure of CLAD following heating treatment and the fibrous CLAD structures could be clearly detected.

### 3.2. DSC

**Figure 4:**
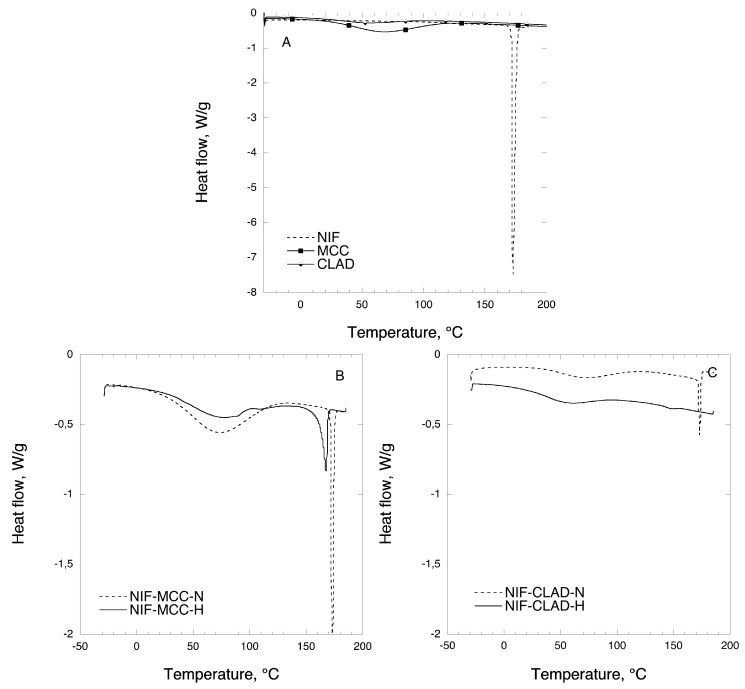
DSC analysis of (**A**) pure NIF, MCC and CLAD, (**B**) NIF-MCC and (**C**) NIF-CLAD 20% *w*/*w* mixtures.

Figure 4 shows the representative DSC profiles for the mixtures of NIF with MCC and CLAD. Figure 4A shows the representative DSC profiles for pure NIF, MCC and CLAD. The melting point of NIF is 173 °C as indicated by the sharp endothermic peak in the graph. The USP monograph reports that the melting point of crystalline NIF is 172–174 °C. The broad peak that is visible in the thermographs of MCC and CLAD between 30 and 100 °C corresponds to water evaporation. The events associated with the moisture evaporation events in mixtures will not be discussed further in the article as they have been extensively covered in the literature [25,27]. Figure 4B shows the DSC profiles for non-heated (N) and heated (H) mixtures of NIF with MCC. The endothermic peak in the region around 165–175 °C, which is visible in NIF-MCC for both non-heated and heated mixtures, corresponds to the melting of NIF. It is obvious that the peak for the heated mixtures is much smaller and Tm shifts from 173 °C to around 167 °C compared to the non-heated sample. Figure 4C shows the DSC profiles for non-heated and heated mixtures of NIF with CLAD. The endothermic peak of the non-heated NIF mixture with CLAD is almost identical to that of the non-heated mixtures with MCC, which shows that the NIF melts at 173 °C as expected. Interestingly, in the heated mixture of CLAD, a barely visible halo is detected at 149 °C. The absence of a sharp melting endotherm suggests that NIF is probably transformed into an amorphous form in the heated NIF-CLAD mixture.

Table 2 summarizes the results of the DSC analysis for NIF mixtures. From Table 2, we concluded that NIF still largely has a crystalline structure in the mixture with MCC even after heating, i.e., crystallinity index of 97.3% and 52.5% for non-heated and heated mixtures, respectively. Surprisingly, the crystallinity index of NIF in the non-heated mixtures with CLAD is only 35.7%, which suggests that some molecular rearrangement must have occurred during mixing. The crystallinity index of NIF in heated mixture with CLAD is only 3.3% based on the endotherm value for the halo around 149 °C, which suggests strong melting point depression.

### 3.3. X-ray Diffraction

**Figure 5:**
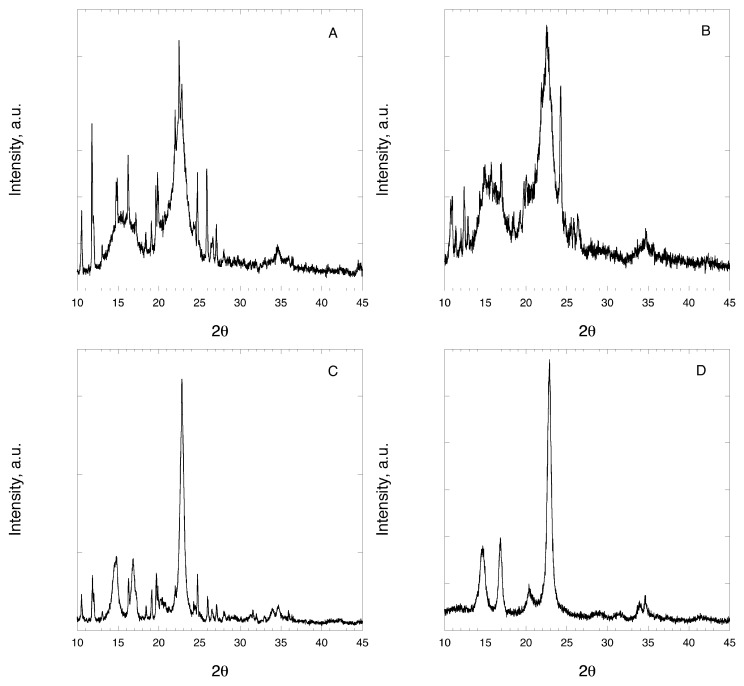
X-ray diffraction images of (**A**) NIF-MCC-N, (**B**) NIF-MCC-H, (**C**) NIF-CLAD-N and (**D**) NIF-CLAD-H 20% *w*/*w* mixtures.

Figure 5 shows the XRD profiles of NIF mixtures with MCC and CLAD. XRD profiles for pure NIF, MCC and CLAD are presented separately in Figure S2 in Supplementary Material. The characteristic peaks for MCC are as follows: broad halo centered around 16°, main peak at around 22° and a minor peak around 34°. The characteristic diffraction peaks for CLAD are sharper and better resolved than those for MCC. In particular, the characteristic peaks were found to be: at 14° (main), 17° (main), 20° (minor), 22° (main) as well as 34° and 35° (both minor). The diffraction profile for NIF is characterized with many sharp and well-resolved peaks over the entire range of studied diffraction angles. 

When studying the XRD profiles of the mixtures, we found that the characteristic sharp peaks for crystalline NIF are clearly seen in both non-heated and heated mixtures of MCC, which are overlaid on the cellulose pattern. These sharp peaks of NIF are also visible in the non-heated mixture of NIF-CLAD. However, they are essentially suppressed in the heated NIF-CLAD mixture. The absence of the characteristic sharp diffraction peaks of NIF suggests that the drug is in the amorphous state, which is consistent with DSC observations. 

Overall, the results of the DSC and XRD analysis suggest that the molecular state of NIF in the heated mixture with CLAD is substantially different from that of the non-heated CLAD mixture as well as those for non-heated and heated mixtures of MCC. The experimental evidence from two independent solid-state characterization methods suggests that NIF is in the amorphous state in the NIF-CLAD-H mixtures, which is expected to result in a higher dissolution rate.

### 3.4. In Vitro Dissolution in Biorelevant Media

**Figure 6:**
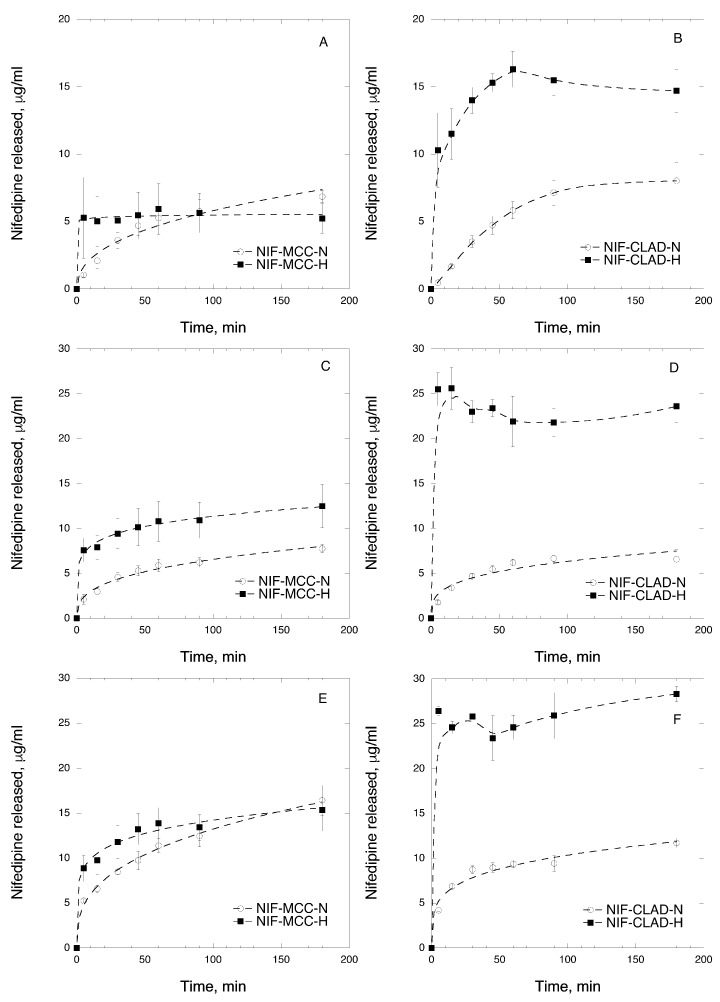
In vitro time-dependent NIF dissolution in biorelevant media: (**A**) NIF-MCC in SGF; (**B**) NIF-CLAD in SGF; (**C**) NIF-MCC in FaSIF; (**D**) NIF-CLAD in FaSIF; (**E**) NIF-MCC in FeSIF and (**F**) NIF-CLAD in FeSIF 20% *w*/*w* mixtures. The results are the average of three measurements, with the standard deviation shown as error bars. The dashed line is added as a visual guide.

In order to investigate the dissolution behavior of NIF in mixtures with cellulose, in vitro dissolution tests in biorelevant media were performed. Figure 6 shows the NIF dissolution curves of non-heated and heated MCC and CLAD mixtures in the respective biorelevant media. Figure 6A,B shows the dissolution rate of NIF in SGF, which has the lowest apparent solubility of NIF. Figure 6A shows that the heated NIF-MCC mixture has a slightly improved dissolution rate compared to the non-heated NIF-MCC mixture. The main difference between these two samples is observed during the first 5 min of the dissolution, in which the heated NIF-MCC mixture has already increased to 5 ± 1 μg/mL compared to only 1 ± 1 μg/mL of the non-heated NIF-MCC mixture at the same time point. It should be noted that both mixtures increased at 6 ± 1 and 5 ± 1 μg/mL, which is the solubility limit of crystalline NIF in SGF. Figure 6B shows the NIF release from CLAD mixtures. The C_max_ of NIF release from NIF-CLAD-N mixture in SGF was relatively higher than that of the NIF-MCC-H sample, leveling at 7 ± 1 μg/mL after 90 min. However, the initial rate of NIF dissolution during the first 15 min for NIF-CLAD-N was not as rapid as that for the NIF-MCC-H mixture and it was more similar to that of NIF-MCC-N. The release of NIF from the heated CLAD mixture showed an interesting profile. The amount of released drug for NIF-CLAD-H sample was almost 2 times higher after 5 min and 2.7 times higher after 45 min than that for NIF-MCC-H at similar time points. Compared to the non-heated MCC and CLAD mixtures, after 5 min, the release of NIF from NIF-CLAD-H was nearly 20 times more NIF. After 45 min, a C_max_ value of 16 ± 1 μg/mL was achieved in NIF-CLAD-H after which it gradually decreased to 14 ± 2 μg/mL, where it remained for the rest of experiment. Apparently, NIF remained in the supersaturated state throughout the entire experiment. It should be noted that the C_max_ for NIF-CLAD-H in SGF corresponds to 40% of the total loaded dose.

Figure 6C,D shows the dissolution profiles of NIF from cellulose mixtures studied in FaSIF. As expected, the apparent solubility of NIF in SIF was substantially higher than that in SGF due to the higher pH of SIF. The apparent solubility of NIF in FaSIF was greater in the heated mixtures compared to the non-heated ones, both for MCC and CLAD. In general, the apparent solubility plateau level for the heated samples was achieved faster than that for the non-heated mixtures. The C_max_ in FaSIF for NIF-MCC-N and NIF-MCC-H was 7.7 ± 0.5 and 12.4 ± 2.4 μg/mL, respectively. On the other hand, the C_max_ in FaSIF for NIF-CLAD-N and NIF-CLAD-H was 6.6 ± 1 and 25.6 ± 1.7 μg/mL, respectively. Thus, the C_max_ in FaSIF for NIF-CLAD-H was two times higher than that for NIF-MCC-H. Furthermore, this high amount of dissolved NIF was achieved within the first 15 min of dissolution in FaSIF for NIF-CLAD-H, which is nearly 7.7 times higher than that for NIF-CLAD-N sample at a similar time point. It should also be noted that even at the highest level of dissolution observed in the NIF-CLAD-H sample, the amount of released NIF was only 64% of the total loaded dose.

Figure 6E,F shows the dissolution profiles of NIF from cellulose mixtures studied in FeSIF. In FeSIF, the observed rate of NIF dissolution was much faster for the heated mixtures compared to the non-heated ones. The C_max_ in FeSIF for NIF-MCC-N and NIF-MCC-H was 16.4 ± 1.7 and 15.3 ± 2.3 μg/mL, respectively. On the other hand, the C_max_ in FeSIF for NIF-CLAD-N and NIF-CLAD-H was 11.7 ± 0.5 and 28.3 ± 0.9 μg/mL, respectively. Thus, the C_max_ in FeSIF for NIF-CLAD-H was 1.8 times higher than that for NIF-MCC-H, which is comparable to that in FaSIF. Furthermore, this high amount of dissolved NIF was achieved within the first 5 min of dissolution in FeSIF for NIF-CLAD-H, after which it fluctuated between 25 and 28 μg/mL for the rest of the experiment. The amount of NIF dissolved during the first 5 min of the experiment was nearly 6 times higher than that for NIF-CLAD-N mixture at a similar time point. It should also be noted that even at the highest level of dissolution observed in the NIF-CLAD-H sample, the amount of released NIF was only 70% of the total loaded dose.

Overall, there seems to be a significant improvement in the apparent solubility and dissolution properties of NIF in heated CLAD mixtures in all tested biorelevant media due to amorphization of the drug.

### 3.5. Solid-State Stability

**Figure 7:**
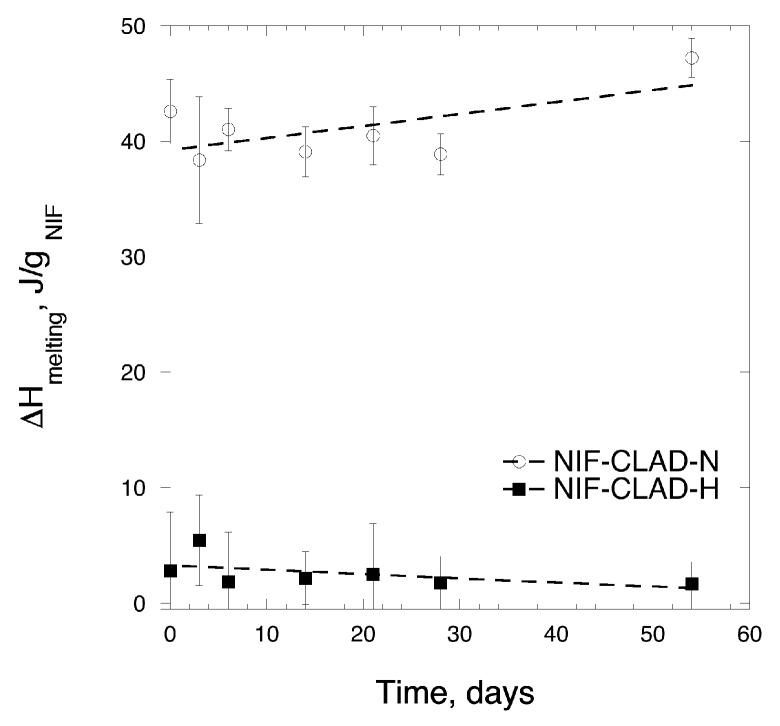
Enthalpy of melting for NIF-CLAD-N and NIF-CLAD-H samples. The dashed line is added as a visual guide.

In order to confirm that NIF remains amorphous in NIF-CLAD-H mixture over a sufficiently long period of time, solid-state stability studies were performed by storing the NIF-CLAD-N and NIF-CLAD-H mixtures at 40 °C and 75% RH. Figure 7 shows the enthalpies of NIF melting during storage. The results of experiments revealed that the amorphous NIF-CLAD-H samples were stable over a period of at least 54 days and no recrystallization of NIF was detected under the experimental conditions.

## 4. Discussion

In this work, we showed an enhancement of the solubility and dissolution rate of NIF with heated CLAD mixtures. NIF supersaturation was achieved, which remained relatively stable for at least 3 h. For amorphous solid dispersions, it is common to observe the so-called spring-and-parachute effect in which the solubility of the drug rapidly reaches transient supersaturation level before gradually declining to the thermodynamically stable equilibrium solubility level. Because the supersaturated solubility of NIF remained relatively stable over the period of experiment, it could be speculated that CLAD has the ability to prevent the re-crystallization of NIF. However, this effect requires further investigation. 

The amorphization of NIF upon mixing and heating with high surface area nanocellulose is associated with the observed effect. A similar effect was previously described for NSAIDs from profens class [30], which suggests that high surface area cellulose possesses largely unexplored potential for improving the bioavailability of poorly soluble substances from various pharmacological classes. From the mechanistic point of view, several authors suggested that aromatic organic molecules have a high affinity for hydrophobic interactions with cellulose [17,32,33,34].

It has been reported in literature that NIF shows pH-independent solubility at the physiological pH of GIT. In this work, we observed that NIF solubility in SGF was lower than that in FaSIF and FeSIF. However, it should be noted that the pH-independent solubility data of NIF has mainly be obtained for SGCs at various pH values and there is a limited number of available articles that focus on its dissolution in various buffer solutions [10]. It should be noted that NIF-CLAD-H formulation showed comparable, if not higher, solubility in SGF as that reported earlier [28]. However, in FaSIF and FeSIF, the observed values were nearly 60% higher than in SGF.

The facile method of IR NIF formulation with nanocellulose opens new possibilities for designing directly compressed tablets with enhanced PK properties as an alternative to traditional SGCs. It has been reported that IR NIF SCGs experience a deviation from a linear correlation between AUCs and doses at a higher drug content, i.e., 20 mg vs. 5–10 mg. In particular, the mean dose-normalized C_max_ was much lower and t_max_ was longer than expected for the highest dose, i.e., 20 mg [35]. Accordingly, it was proposed that the delay in NIF absorption could be due to the precipitation of NIF from the SGCs when liberated in the fasted stomach. Following transport along the gastro-intestinal tract, the precipitated NIF would re-dissolve in the small intestine and then be absorbed in a lower part of the small intestine, leading to a delay in the time needed to reach C_max_ and the decelerated elimination phase [36]. Thus, the rapid dissolution and enhanced NIF solubility that was observed in biorelevant media is promising but should further be investigated in extensive PK studies for their potential effects on C_max_ and t_max_ values compared to commercially available NIF SGCs for bioequivalence. If the new dosage form is not bioequivalent to SGCs, this may lead to concentrations either below or above the recommended therapeutic window. Thus, since the BCS-based biowaiver procedure for NIF cannot be recommended for either the SGC or tablet IR formulations, bioequivalence needs to be ensured in vivo [10].

## 5. Conclusions

An enhanced solubility and faster dissolution rate of NIF were observed in NIF-CLAD-H mixtures in all tested biorelevant media, i.e., SGF, FaSIF and FeSIF. The observed enhancement of solubility and dissolution was due to amorphization of NIF in high surface area nanocellulose powder. Ordinary MCC failed to produce an enhancement of a similar magnitude. Amorphous NIF-CLAD-H mixtures were stable under 40 °C and 75% RH conditions for at least 54 days, when the study was halted. The results of the study suggest that dry powder formulation using high surface area nanocellulose with heat is a facile new strategy for formulating calcium channel blocker drugs, which could be an alternative to currently used SGCs. Future studies should focus on determining their bioequivalence to NIF SGCs to further elucidate the clinical potential of the platform.

## Figures and Tables

**Table 1:** Chemical structure of NIF.

Drug	Structure	IUPAC Name
NIF	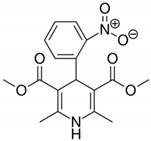	1,4-dihydro-2,6-dimethyl-4-(2-nitrophenyl)-3,5-pyridine dicarboxylic acid dimethyl ester

**Table 2:** Summary of DSC analysis of 20% *w*/*w* NIF mixtures with cellulose. The results are the average of three measurements, with the standard deviation also provided.

	T_on_, °C	T_m_, °C	ΔH, J/g _mix_	CrI *, %
NIF	172.1 ± 0.1	172.8 ± 0.1	105.4 ± 2.2	100
NIF-MCC-N	171.7 ± 0.0	173.1 ± 0.1	20.5 ± 0.8	97.4
NIF-MCC-H	162.6 ± 1.4	167.8 ± 0.8	11.1 ± 1.9	52.5
NIF-CLAD-N	171.8 ± 0.0	173.1 ± 0.1	7.5 ± 2.3	35.5
NIF-CLAD-H	142.4 ± 1.8	148.7 ± 1.9	0.7 ± 0.5	3.3

* Calculated according to Equation (2).

## Supplementary Materials

The following are available online at http://www.mdpi.com/1999-4923/11/1/37/s1, Figure S1: Thermogravimetric analysis (TGA) profile of NIF maintained at 174 °C for 1 h. Figure S2: XRD profiles of MCC (A), CLAD (B) and NIF (C).
