# Supplementary material for: Immediate-Release Nifedipine Binary Dry Powder Mixtures with Nanocellulose Featuring Enhanced Solubility and Dissolution Rate
Source: Pharmaceutics. 2019 Jan 18;11(1):37. doi: 10.3390/pharmaceutics11010037 (PMC6359467; doi:10.3390/pharmaceutics11010037)
Supplement: Supplementary file 1 [file pharmaceutics-11-00037-s001.pdf]

pharmaceutics

Supplementary Materials: Immediate-Release Nifedipine Binary Dry Powder Mixtures with Nanocellulose Featuring Enhanced Solubility and Dissolution Rate

Athanasios Mantas and Albert Mihranyan *

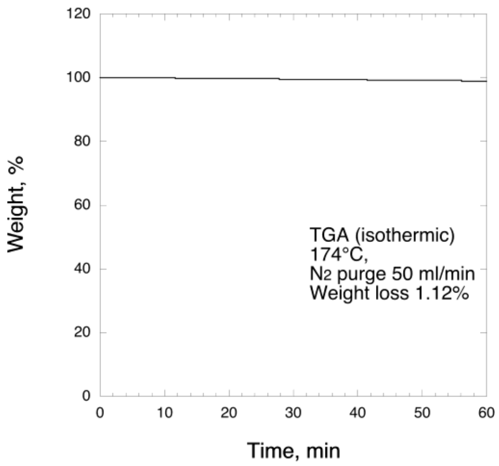

Figure 1. Thermogravimetric analysis (TGA) profile of NIF maintained at 174 °C for 1 h.

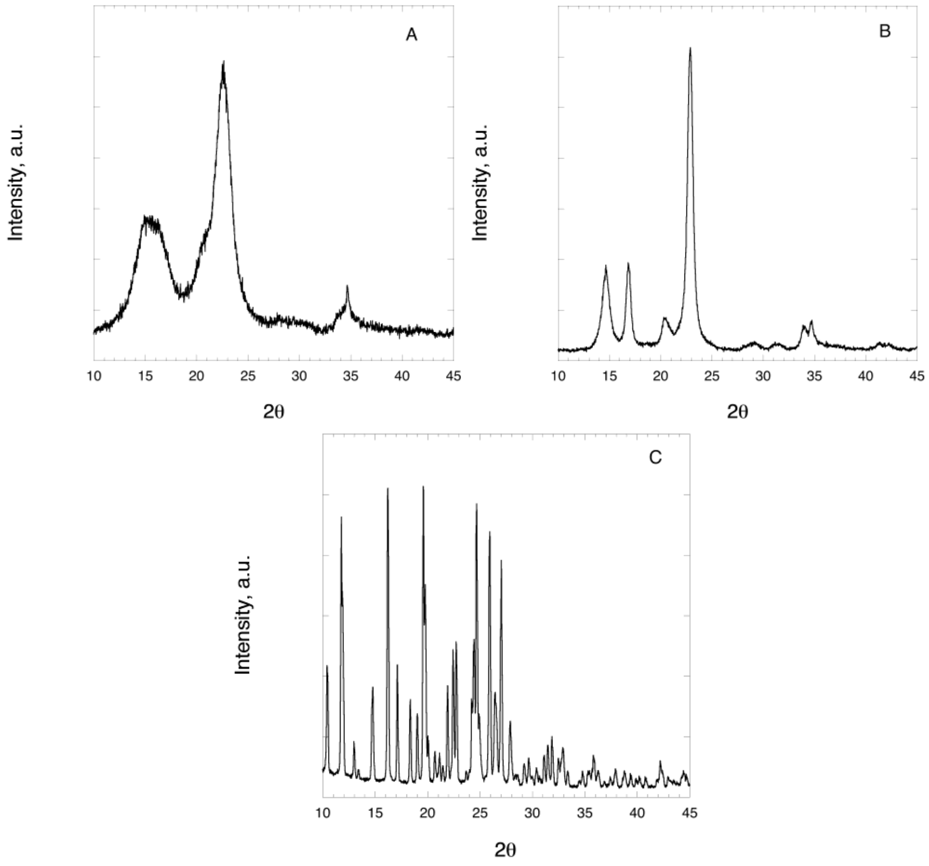

Figure 2. XRD profiles of MCC (**A**), CLAD (**B**) and NIF (**C**).
